# Supplementary material for: Evolution of T cells in the cancer-resistant naked mole-rat
Source: Nat Commun. 2024 Apr 11;15:3145. doi: 10.1038/s41467-024-47264-x (PMC11009300; doi:10.1038/s41467-024-47264-x)
Supplement: Supplementary file 1 — Supplementary Information [file 41467_2024_47264_MOESM1_ESM.pdf]

Evolution of T cells in the cancer-resistant naked mole-rat

Tzuhua D. Lin[1,†,] Nimrod D. Rubinstein[1,†,*], Nicole L. Fong[1], Megan Smith[1], Wendy Craft[1], Baby Martin-McNulty[1], Rebecca Perry[3], Martha A. Delaney[2], Margaret A. Roy[1], Rochelle Buffenstein[1,3,*]

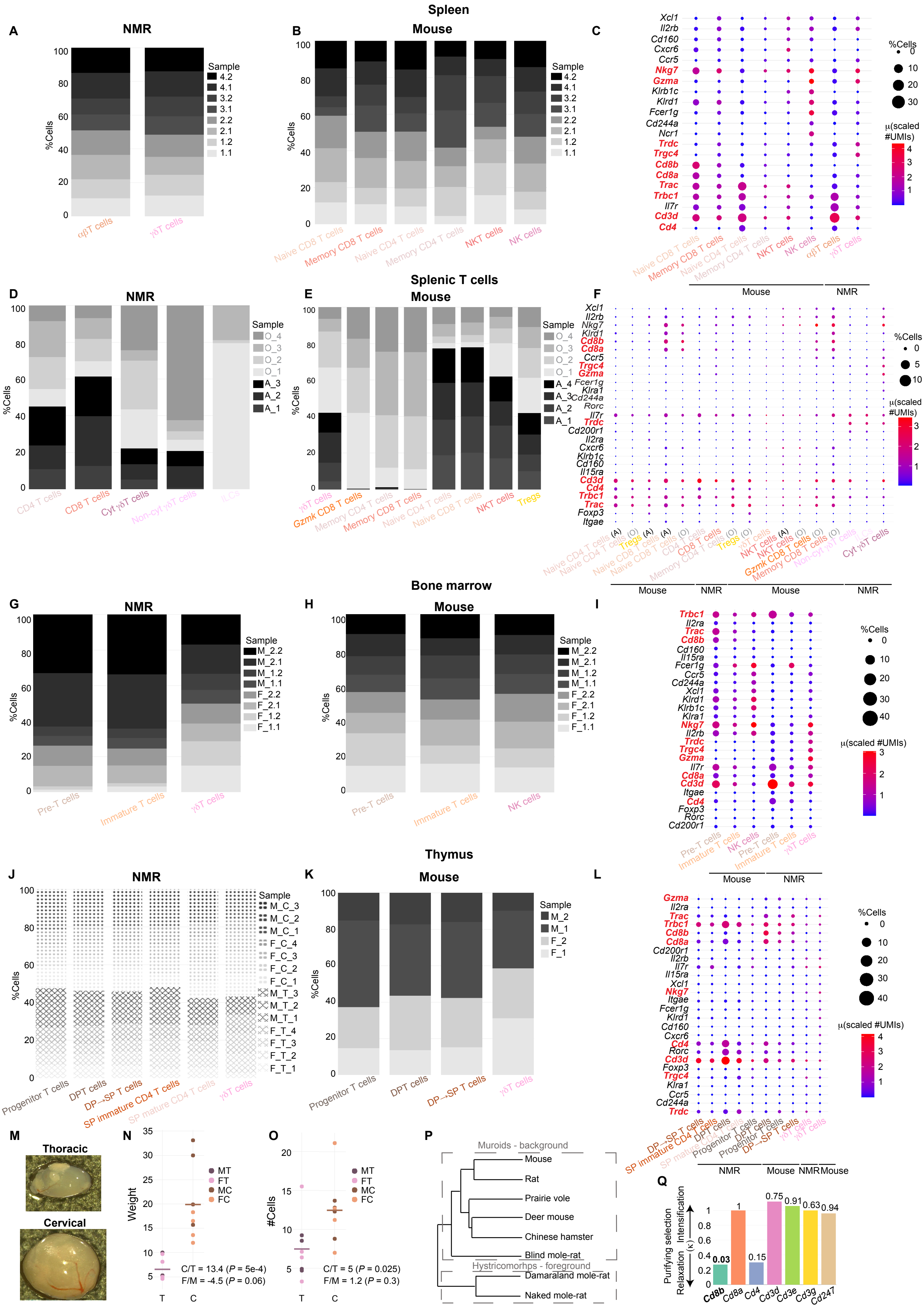

Spleen
A
NMR
%Cells
Sample
4.2
4.1
3.2
3.1
2.2
2.1
1.2
1.1
αβT cells
γδT cells
B
Mouse
Naive CD8 T cells
Memory CD8 T cells
Naive CD4 T cells
Memory CD4 T cells
NKT cells
NK cells
C
%Cells
µ(scaled #UMIs)
Xcl1
Il2rb
Cd160
Cxcr6
Ccr5
Nkg7
Gzma
Klrb1c
Klrd1
Fcer1g
Cd244a
Ncr1
Trdc
Trgc4
Cd8b
Cd8a
Trac
Trbc1
Il7r
Cd3d
Cd4
Mouse
NMR
Splenic T cells
D
NMR
E
Mouse
Sample
O_4
O_3
O_2
O_1
A_4
A_3
A_2
A_1
CD4 T cells
CD8 T cells
Cyt γδT cells
Non-cyt γδT cells
ILCs
γδT cells
Gzmk CD8 T cells
Tregs
F
Rorc
Cd200r1
Il2ra
Klrb1c
Il15ra
Klra1
Foxp3
Itgae
Bone marrow
G
NMR
Sample
M_2.2
M_2.1
M_1.2
M_1.1
F_2.2
F_2.1
F_1.2
F_1.1
Pre-T cells
Immature T cells
γδT cells
H
Mouse
NK cells
I
Thymus
J
NMR
Sample
M_C_3
M_C_2
M_C_1
F_C_4
F_C_3
F_C_2
F_C_1
M_T_3
M_T_2
M_T_1
F_T_4
F_T_3
F_T_2
F_T_1
Progenitor T cells
DPT cells
DP→SP T cells
SP immature CD4 T cells
SP mature CD4 T cells
K
Mouse
Sample
M_2
M_1
F_2
F_1
L
M
Thoracic
Cervical
N
Weight
MT
FT
MC
FC
C/T = 13.4 (P = 5e-4)
F/M = -4.5 (P = 0.06)
T
C
O
#Cells
C/T = 5 (P = 0.025)
F/M = 1.2 (P = 0.3)
P
Muroids - background
Mouse
Rat
Prairie vole
Deer mouse
Chinese hamster
Blind mole-rat
Hystricomorphs - foreground
Damaraland mole-rat
Naked mole-rat
Q
Purifying selection
Relaxation
Intensification
(κ)
0.03
1
0.15
0.75
0.91
0.63
0.94
Cd8b
Cd8a
Cd4
Cd3d
Cd3e
Cd3g
Cd247

Fig. S1: ScRNA-seq of circulating T-cell subsets reveals that the NMR has a large population of γδT cells

Bar-charts of the cell proportions assigned to each NK and T -cell subset from spleens of (**A**) four NMRs and (**B**) four mice (two technical replicates for each sample in each species). Bars are relatively uniformly represented by all samples; **(C)** gene-by-cell-subset heatmap of the NK and T -cell subsets of the two species. Bar-charts of the cell proportions assigned to each T-cell subset from spleens of (**D**) three adult and four old NMRs and (**E**) four adult and four old mice. NMR ILC and the mouse CD4 and CD8 subsets are non-uniformly represented by the samples; **(F)** gene-by-cell-subset heatmap of the T-cell subsets of the two species. Bar-charts of the cell proportions assigned to each T-cell subset from bone marrows of (**G**) two male and two female NMRs and (**H**) two male and two female mice (two technical replicates for each sample in each species). Bars are relatively uniformly represented by all samples; **(I)** gene-by-cell-subset heatmap of the T-cell subsets of the two species. Bar-charts of the cell proportions assigned to each T-cell subset from thymuses of (**J**) seven NMRs (thoracic and cervical) and (**K**) four mice (thoracic). Bars are relatively uniformly represented by all samples; **(L)** gene-by-cell-subset heatmap of the T-cell subsets of the two species. Heatmaps are color-coded by expression levels and diameter-coded by the proportion of the gene-expressing cells in the cell subset. Genes identifying γδT-cell subsets are labeled in red. Comparison between the NMR thoracic and cervical thymic tissues showing that the cervical tissue is larger in **(M)** size; **(N)** weight; and **(O)** number of cells. Quantification of the intensity of purifying selection operating on **(P)** hystricomorph relative to muroid **(Q)** T-cell co-receptor genes, revealing the significant relaxation of purifying selection in the hystricomorph *Cd8b*. Values above bars are multiple-hypothesis-adjusted *p* values of the hystricomorph vs. muroid relaxation in purifying selection (RELAX; Methods). **Abbreviations**: A – Adult; C – Cervical; Cyt – Cytotoxic; DP – Double Positive; F – Female; M – Male; NK – Natural Killer; NKT – Natural Killer T; Non-cyt – Non-cytotoxic; O – Old; P – P-value; SP – Single Positive; T – Thoracic. Source data are provided as source data file.

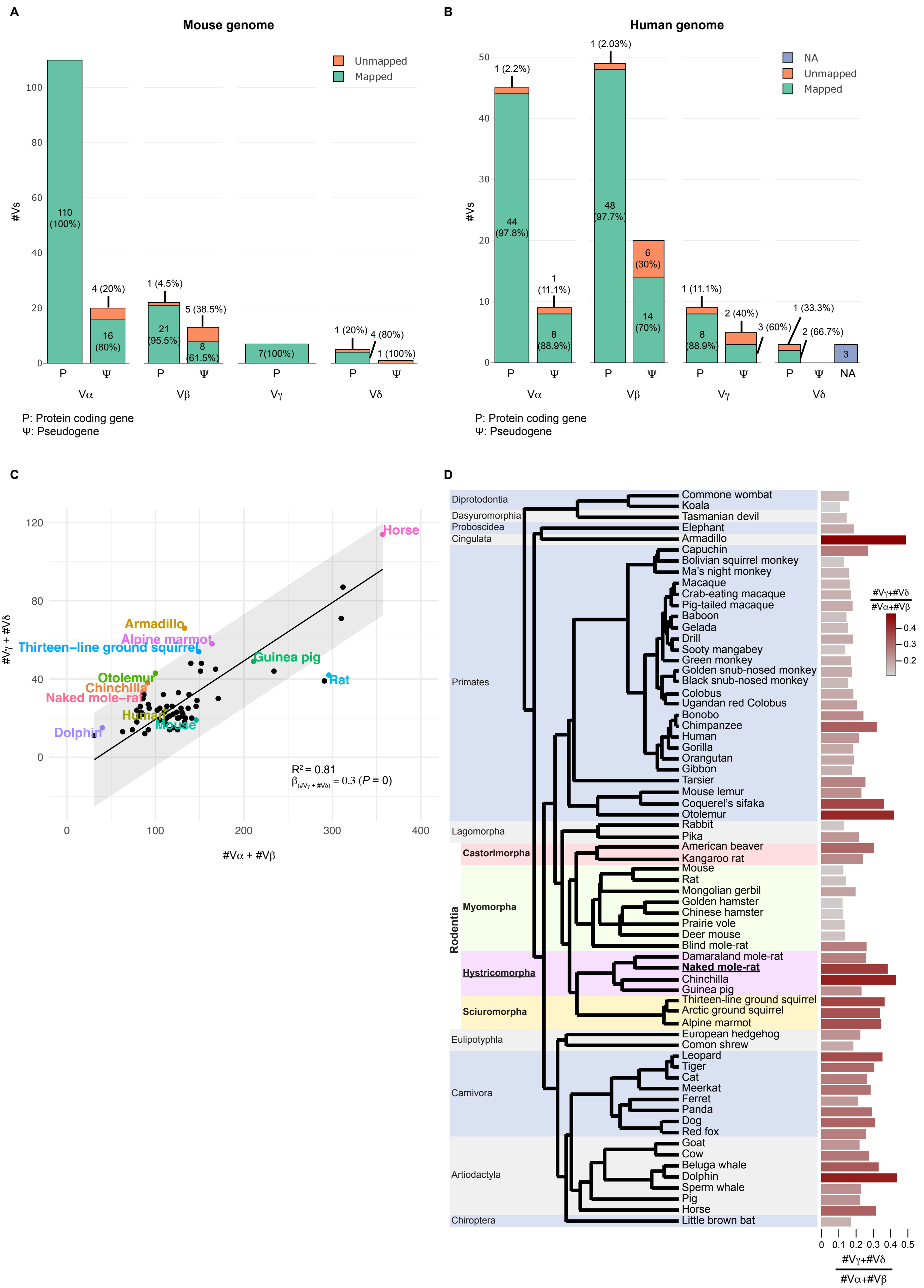
A
Mouse genome
Unmapped
Mapped
#Vs
110 (100%)
4 (20%)
16 (80%)
1 (4.5%)
21 (95.5%)
5 (38.5%)
8 (61.5%)
7(100%)
1 (20%)
4 (80%)
1 (100%)
P Ψ P Ψ P P Ψ
Vα Vβ Vγ Vδ
P: Protein coding gene
Ψ: Pseudogene
B
Human genome
NA
Unmapped
Mapped
1 (2.2%)
44 (97.8%)
1 (11.1%)
8 (88.9%)
1 (2.03%)
48 (97.7%)
6 (30%)
14 (70%)
1 (11.1%)
8 (88.9%)
2 (40%)
3 (60%)
1 (33.3%)
2 (66.7%)
3
P Ψ P Ψ P Ψ P Ψ NA
Vα Vβ Vγ Vδ
P: Protein coding gene
Ψ: Pseudogene
C
#Vγ + #Vδ
#Vα + #Vβ
Horse
Armadillo
Alpine marmot
Thirteen–line ground squirrel
Guinea pig
Rat
Otolemur
Chinchilla
Naked mole–rat
Human
Mouse
Dolphin
R² = 0.81
β(#Vγ + #Vδ) = 0.3 (P = 0)
D
Diprotodontia
Dasyuromorphia
Proboscidea
Cingulata
Primates
Lagomorpha
Castorimorpha
Myomorpha
Rodentia
Hystricomorpha
Sciuromorpha
Eulipotyphla
Carnivora
Artiodactyla
Chiroptera
Commone wombat
Koala
Tasmanian devil
Elephant
Armadillo
Capuchin
Bolivian squirrel monkey
Ma's night monkey
Macaque
Crab-eating macaque
Pig-tailed macaque
Baboon
Gelada
Drill
Sooty mangabey
Green monkey
Golden snub-nosed monkey
Black snub-nosed monkey
Colobus
Ugandan red Colobus
Bonobo
Chimpanzee
Human
Gorilla
Orangutan
Gibbon
Tarsier
Mouse lemur
Coquerel's sifaka
Otolemur
Rabbit
Pika
American beaver
Kangaroo rat
Mouse
Rat
Mongolian gerbil
Golden hamster
Chinese hamster
Prairie vole
Deer mouse
Blind mole-rat
Damaraland mole-rat
Naked mole-rat
Chinchilla
Guinea pig
Thirteen-line ground squirrel
Arctic ground squirrel
Alpine marmot
European hedgehog
Comon shrew
Leopard
Tiger
Cat
Meerkat
Ferret
Panda
Dog
Red fox
Goat
Cow
Beluga whale
Dolphin
Sperm whale
Pig
Horse
Little brown bat
#Vγ+#Vδ / #Vα+#Vβ

Fig. S2: Mammalian phyletic pattern of TCR regions reveals large diversity of γ and δ, relative to α and β, variable regions in the NMR genome

Benchmarking the detection of variable TCR regions in 67 mammalian genomes using the **(A)** mouse and **(B)** human genomes and their corresponding annotations where each figure shows the number annotated variable regions, from all four TCR loci, that were mapped, unmapped, and uniquely found (NA) by our approach (Methods), revealing a low false detection rate; **(C)** the association between the sum of γ and δ and the sum of α and β variable TCR regions and the strong fit of the phylogenetic least squares model to these data, which is why the diversity of γ and δ variable TCR regions relative to α and β variable TCR regions is more robustly expressed as the residuals of that fit than **(D)** the ratio of the two displayed to the side of the phyletic pattern of 67 mammalian genomes (Methods), color-coded by orders, where the Rodentia order is further color-coded by suborder. **Abbreviations**: V – Variable. Source data are provided as source data file.

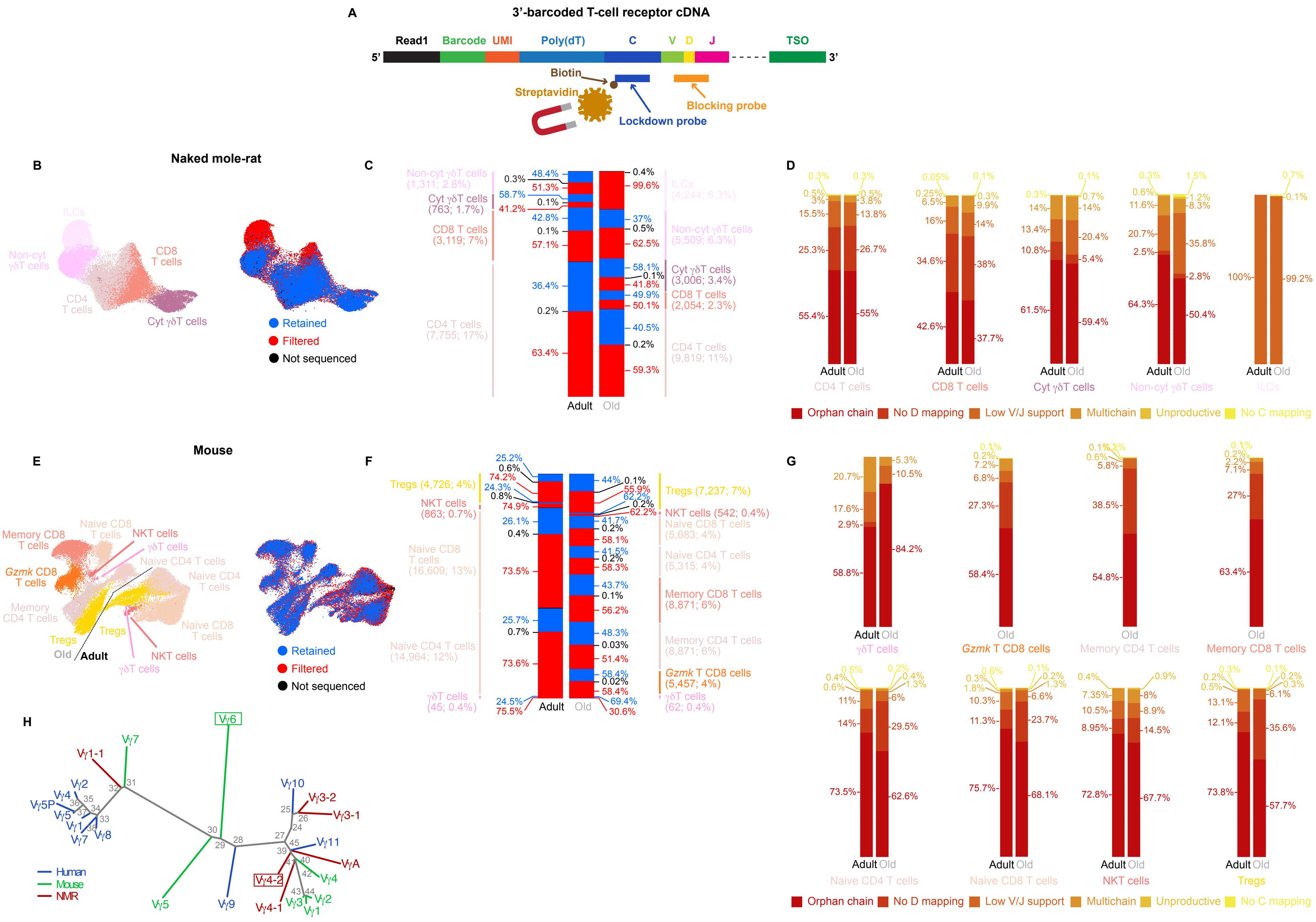
A
3'-barcoded T-cell receptor cDNA
Read1
Barcode
UMI
Poly(dT)
C
V
D
J
TSO
5'
3'
Biotin
Streptavidin
Lockdown probe
Blocking probe
B
Naked mole-rat
ILCs
Non-cyt γδT cells
CD8 T cells
CD4 T cells
Cyt γδT cells
Retained
Filtered
Not sequenced
C
Adult
Old
D
Orphan chain
No D mapping
Low V/J support
Multichain
Unproductive
No C mapping
E
Mouse
Memory CD8 T cells
Naive CD8 T cells
NKT cells
γδT cells
Naive CD4 T cells
Gzmk CD8 T cells
Memory CD4 T cells
Tregs
Old
Adult
F
Tregs (4,726; 4%)
NKT cells (863; 0.7%)
Naive CD8 T cells (16,609; 13%)
Naive CD4 T cells (14,964; 12%)
γδT cells (45; 0.4%)
Tregs (7,237; 7%)
NKT cells (542; 0.4%)
Naive CD8 T cells (5,683; 4%)
Naive CD4 T cells (5,315; 4%)
Memory CD8 T cells (8,871; 6%)
Memory CD4 T cells (8,871; 6%)
Gzmk T CD8 cells (5,457; 4%)
γδT cells (62; 0.4%)
G
H
Vγ1-1
Vγ7
Vγ6
Vγ2
Vγ4
Vγ5P
Vγ5
Vγ1
Vγ7
Vγ8
Vγ10
Vγ3-2
Vγ3-1
Vγ11
VγA
Vγ4-2
Vγ4
Vγ2
Vγ3
Vγ1
Vγ4-1
Vγ9
Vγ5
Human
Mouse
NMR

Fig. S3: Single-cell TCR sequencing reveals surprising features of clonotypic diversity in naked mole-rat T-cell subsets

(A) An illustration of the hybridization-capture approach that we developed in order to obtain full-length single-cell TCR sequences utilizing previously generated 10× 3'-barcoded samples (Methods); **(B)** UMAP projections of T cells from spleens of three adult and four old NMRs. Each point represents a cell, the left UMAP is color-coded by T-cell subset and the right UMAP is color-coded by whether their corresponding hybridization-capture sequenced reads were retained, filtered, or not sequenced at all; the stacked bar-charts in **(C)** provide these retention/filtering proportions per each T-cell subset; the stacked bar-charts in **(D)** break down the proportions of filtered hybridization-capture sequenced reads by either one of the six filtering criteria we used (Methods) per each T-cell subset; **(E-G)** are figures corresponding to Fig. S3B-D, for T cells from spleens from the four adult and four old mice; **(H)** phylogenetic reconstruction of the mammalian variable γ TCR regions, pruned to only show the human, mouse, and NMR leaves (Methods). Bootstrap support values (%) are labeled at each split and the boxed leaves are the variable γ TCR regions that comprise the dominant clonotypes found in the NMR and mouse γδT-cell subsets. **Abbreviations**: C – Constant; Cyt – Cytotoxic; D – Diversifying; J – Joining; Non-cyt – Non-cytotoxic. Source data are provided as source data file.

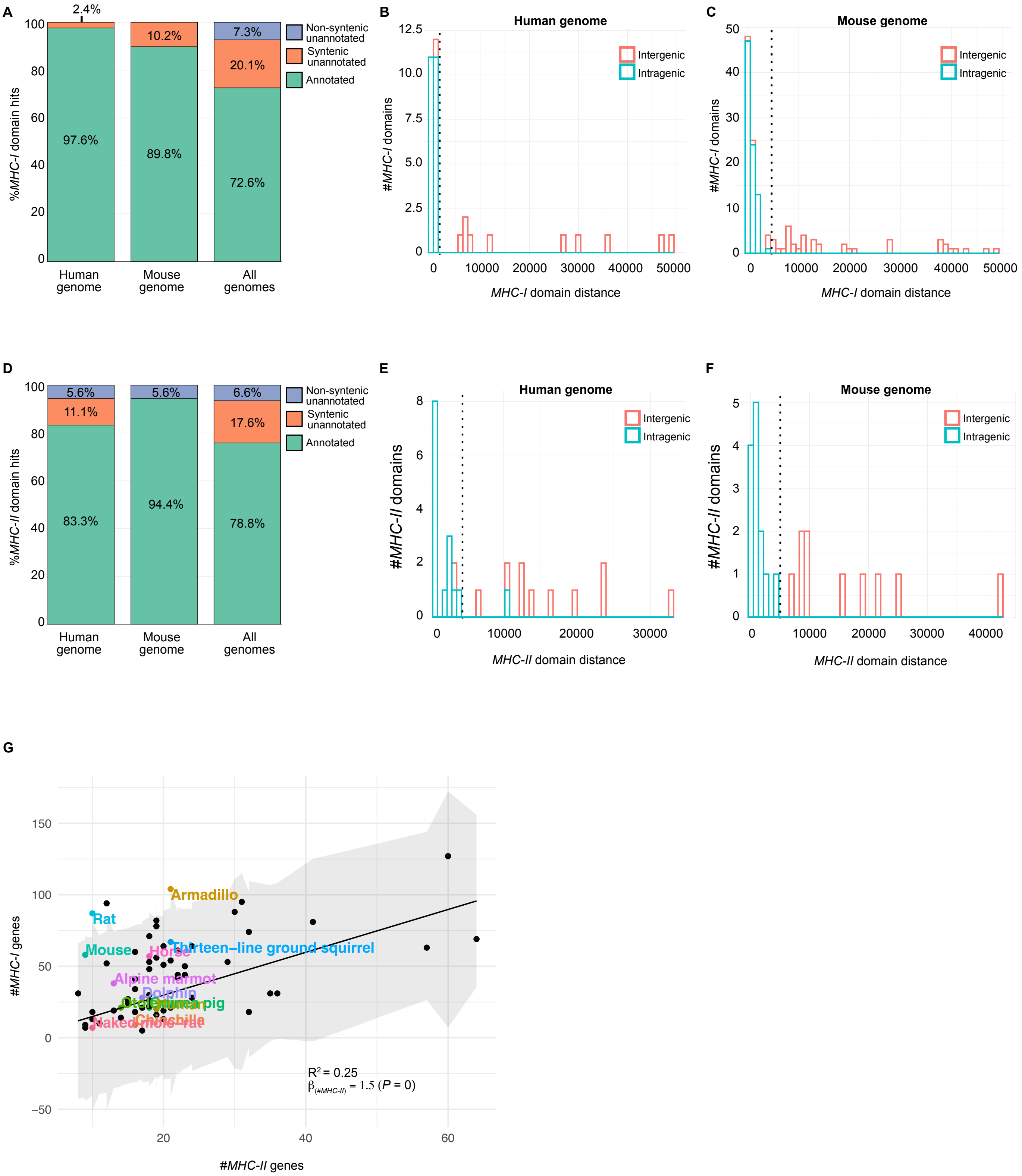
A
B
C
D
E
F
G
2.4%
97.6%
10.2%
89.8%
7.3%
20.1%
72.6%
%MHC-I domain hits
Human genome
Mouse genome
All genomes
Non-syntenic unannotated
Syntenic unannotated
Annotated
Human genome
Mouse genome
Intergenic
Intragenic
#MHC-I domains
MHC-I domain distance
5.6%
11.1%
83.3%
5.6%
94.4%
6.6%
17.6%
78.8%
%MHC-II domain hits
#MHC-II domains
MHC-II domain distance
Armadillo
Rat
Mouse
Horse
Thirteen–line ground squirrel
Alpine marmot
Dolphin
#MHC-I genes
#MHC-II genes
R² = 0.25
β(#MHC-II) = 1.5 (P = 0)

Fig. S4: Mammalian phyletic pattern of *MHC* gene families reveals a small *MHC-I*, relative to *MHC-II*, gene-family size in the naked mole-rat genome

Benchmarking the detection of *MHC-I* protein-coding and pseudo -genes, and unannotated putative pseudogenes in 67 mammalian genomes. **(A)** stacked bar-chart showing the proportion of *MHC-I* domain hits that are annotated, unannotated but are syntenic to annotated *MHC-I* genes, and unannotated and not syntenic to annotated *MHC-I* genes, where the latter two categories form an upper bound to the false positive rate of our approach (Methods). Distributions of genomic distances between *MHC-I* domains, either of the same gene or genomically adjacent genes, in the **(B)** human and **(C)** mouse genomes (using only annotated *MHC-I* genes). Vertical dotted black lines represent the genomic distance cutoff below which any pair of genomically adjacent *MHC-I* domain hits were assigned to the same *MHC-I* gene. **(D-F)** figures corresponding to (A-C) for *MHC-II* genes; **(G)** the association between the number of *MHC-I* and *MHC-II* genes and the weak fit of the phylogenetic least squares model to these data, which is why the ratio between the two was used to express the relative diversity of *MHC-I* (Fig. 4B), rather than the residuals of the model, as applied to the variable TCR regions. **Abbreviations**: P – P-value. Source data are provided as source data file.

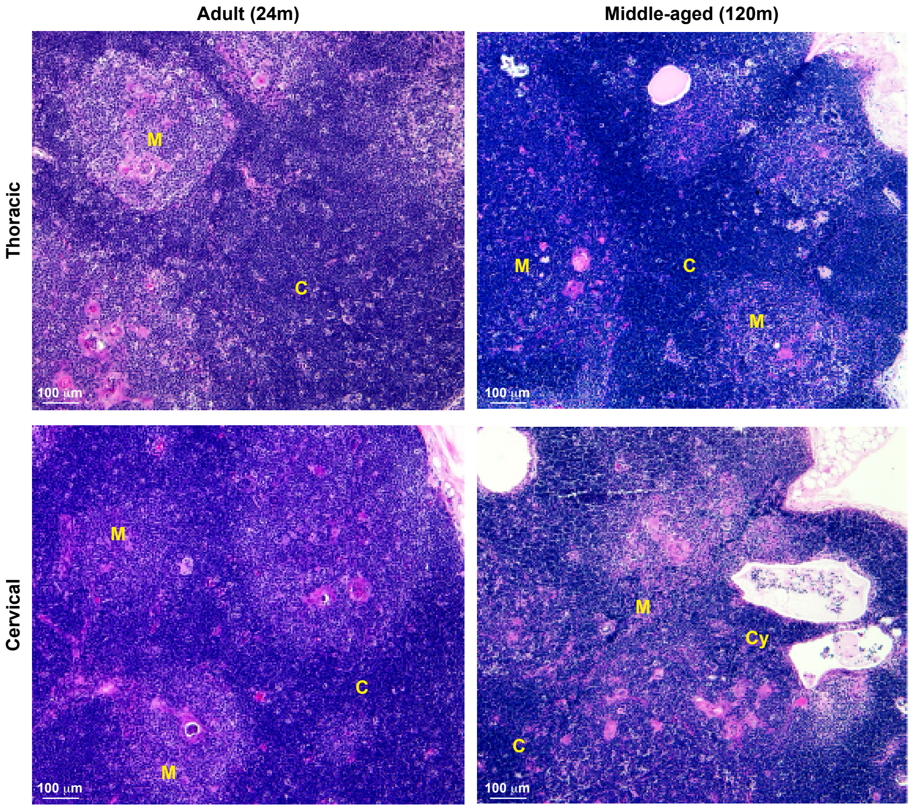
Adult (24m)
Middle-aged (120m)
Thoracic
Cervical
M
C
M
C
M
M
M
C
M
Cy
C
100 µm
100 µm
100 µm
100 µm

Fig. S5: Thoracic and cervical middle-age NMR thymuses show equivalent microscopic signs indicative of involution

Images of hematoxylin and eosin (H&E) stained sections from thoracic (top) and cervical (bottom) thymic tissues of adult (left) and middle-aged (right) NMRs, showing that at middle age, both thymic tissues present similar signs of indicative of involution, including reduced contrast between cortex and medulla and formation of cysts. **Abbreviations:** C – Cortex; Cy – Cyst; M – Medulla.
