# Supplementary material for: Evolution of T cells in the cancer-resistant naked mole-rat
Source: Nat Commun. 2024 Apr 11;15:3145. doi: 10.1038/s41467-024-47264-x (PMC11009300; doi:10.1038/s41467-024-47264-x)
Supplement: Supplementary file 3 — Description of Additional Supplementary Files [file 41467_2024_47264_MOESM3_ESM.pdf]

Evolution of T cells in the cancer-resistant naked mole-rat

Tzuhua D. Lin[1,†,] Nimrod D. Rubinstein[1,†,*], Nicole L. Fong[1], Megan Smith[1], Wendy Craft[1], Baby Martin-McNulty[1], Rebecca Perry[3], Martha A. Delaney[2], Margaret A. Roy[1], Rochelle Buffenstein[1,3,*]

Data S1: Cell subset marker genes

For each of the cell subsets in each of the scRNA-seq tissues, in each species, the corresponding lists of highly expressed (markers) genes are provided.

Data S2: NMR splenic T-cell clustering data

For each sequenced NMR splenic T cell, the corresponding sample, cell subset, and age are provided.

Data S3: Mouse splenic T-cell clustering data

For each sequenced mouse splenic T cell, the corresponding sample, cell subset, and age are provided.

Data S4: NMR bone marrow clustering data

For each sequenced NMR bone-marrow cell, the corresponding sample, cell subset, and sex are provided.

Data S5: Mouse bone marrow clustering data

For each sequenced mouse bone-marrow cell, the corresponding sample, cell subset, and sex are provided.

Data S6: NMR thymus clustering data

For each sequenced NMR thymocyte, the corresponding sample, cell subset, and thymic tissue are provided.

Data S7: Mouse thymus clustering data

For each sequenced mouse thymocyte, the corresponding sample and cell subset are provided.

Data S8: *Cd8b* multiple codon-sequence alignment

Fasta file of the multiple codon-sequence alignment of the *Cd8b* sequence IDs provided in Data S7.

Data S9: *Cd8a* multiple codon-sequence alignment

Fasta file of the multiple codon-sequence alignment of the *Cd8a* sequence IDs provided in Data S7.

Data S10: *Cd4* multiple codon-sequence alignment

Fasta file of the multiple codon-sequence alignment of the *Cd4* sequence IDs provided in Data S7.

Data S11: *Cd3d* multiple codon-sequence alignment

Fasta file of the multiple codon-sequence alignment of the *Cd3d* sequence IDs provided in Data S7.

Data S12: *Cd3e* multiple codon-sequence alignment

Fasta file of the multiple codon-sequence alignment of the *Cd3e* sequence IDs provided in Data S7.

Data S13: *Cd3g* multiple codon-sequence alignment

Fasta file of the multiple codon-sequence alignment of the *Cd3g* sequence IDs provided in Data S7.

Data S14: *Cd247* multiple codon-sequence alignment

Fasta file of the multiple codon-sequence alignment of the *Cd247* sequence IDs provided in Data S7.

Data S15: Representative sequence information of the *Cd8b, Cd8a, Cd4, Cd3d, Cd3e, Cd3g,* and *Cd247* genes for quantifying relaxation of purifying selection

For each of the genes in each of the species the corresponding gene, transcript, and protein IDs are provided.

Data S16: TCR phyletic pattern

For each of the 67 mammalian genomes, the corresponding chromosome, start position, end position, strand, TCR locus, hit conserved domain, annotated gene ID, annotated gene name, biotype, and an indicator if the hit overlaps an annotated gene are provided.

Data S17: NMR TCR-seq data

For each TCR-sequenced NMR splenic T cell, the corresponding sample, age, cell subset, filter status, filtering reason, and assigned clonotype are provided.

Data S18: Mouse TCR-seq data

For each TCR-sequenced mouse splenic T cell, the corresponding sample, age, cell subset, filter status, filtering reason, and assigned clonotype are provided.

Data S19: *MHC* phyletic pattern

For each of the 67 mammalian genomes used for the phyletic patterns, the corresponding chromosome, start position, end position, strand, *MHC* gene family, hit conserved domain, annotated gene ID, annotated gene name, biotype, and an indicator if the hit overlaps an annotated gene are provided.

Data S20: Thymus age trajectory

For each mouse and NMR sample, the corresponding age (months), sex, body weight (gr), thoracic thymus weight (mg), and NMR cervical thymus weight (mg) are provided.

Data S21: Animals used in this study

For each mouse and NMR animal sample, the corresponding colony (NMRs) or strain (mice), chip ID (NMRs), sex, age (months), tissue, and flags indicating if the sample was used for scRNA-seq, hybridization-capture TCR-seq, and thymus age trajectory analyses are provided.

Data S22: Mammalian genomes used for the phyletic pattern analyses

For each mammalian species, the corresponding taxanomic group, taxanomic group level, Linnaeus naming, genus, epithet, taxon ID, genome assembly, genome annotation source, genome annotation release, Ensembl genome annotation file name, Ensembl genome sequence file name, Ensembl proteome sequence file name, Ensembl transcriptome cDNA sequence file name, Ensembl transcriptome ncRNA sequence file name, genome assembly level, RefSeq genome assembly accession, genome sequencing technology, genome assembler, genome

coverage, RefSeq genome annotation file name, RefSeq genome sequence file name, RefSeq proteome sequence file name, RefSeq RNA sequence file name, RefSeq genome assembly report file name, Ensembl-to-Entrez mapping file name, Ensembl-to-RefSeq mapping file name, and flags indicating if the Ensembl and Refseq genome annotations and genome assemblies were merged are provided.

Data S23: Gene family conserved protein domains

For each gene family, the corresponding conserved protein domain code and accession are provided.

Data S24: Orthogroup assignment

For each orthogroup ID, the corresponding species, gene ID, and gene name are provided.

Data S25: Filtered non-*MHC-I* genomic hit intervals

For each species genome, the corresponding chromosome, start, end, and potential non-*MHC-I* gene family corresponding to a non-*MHC-I* filtered genomic interval are provided.

Data S26: Mouse and NMR hybridization-capture lockdown probe information

For each of the constant regions in the four TCR loci for each species, the corresponding lockdown probe sequence name, the nucleic-acid sequence, the chromosome, relative start position, relative end position, strand, gene ID, gene name, transcript ID, orthologous NMR gene ID, %GC, number of genomic hits, and score are provided.

Data S27: Genomic coordinates of constant TCR regions

For each of the constant regions in the four TCR loci of both NMR and mouse, the corresponding chromosome, start site, end site, and strand are provided.

Data S28: Mouse variable TCR region IgBLAST database

Fasta file of mouse variable TCR region nucleic-acid sequences used as database for IgBLAST.

Data S29: Mouse diversity TCR region IgBLAST database

Fasta file of mouse diversity TCR region nucleic-acid sequences used as database for IgBLAST.

Data S30: Mouse joining TCR region IgBLAST database

Fasta file of mouse joining TCR region nucleic-acid sequences used as database for IgBLAST.

Data S31: NMR variable TCR region IgBLAST database

Fasta file of NMR variable TCR region nucleic-acid sequences used as database for IgBLAST.

Data S32: NMR diversity TCR region IgBLAST database

Fasta file of NMR diversity TCR region nucleic-acid sequences used as database for IgBLAST.

Data S33: NMR joining TCR region IgBLAST database

Fasta file of NMR joining TCR region nucleic-acid sequences used as database for IgBLAST.

Data S34: Mouse germline auxiliary IgBLAST database

Mouse germline auxiliary file used by IgBLAST.

Data S35: NMR germline auxiliary IgBLAST database

NMR germline auxiliary file used by IgBLAST.

Data S36: GUIDENCE2-filtered variable TCRγ multiple amino-acid sequence alignment

Fasta file of the GUIDENCE2-filtered variable TCRγ multiple amino-acid sequence alignment used for building a variable TCRγ phylogenetic tree.
