# Supplementary material for: Evolution of T cells in the cancer-resistant naked mole-rat
Source: Nat Commun. 2024 Apr 11;15:3145. doi: 10.1038/s41467-024-47264-x (PMC11009300; doi:10.1038/s41467-024-47264-x)
Supplement: Supplementary file 40 — Reporting Summary [file 41467_2024_47264_MOESM40_ESM.pdf]

nature portfolio

Corresponding author(s): Nimrod D.Rubinstein
Rochelle Buffenstein

Last updated by author(s): Mar 15, 2024

Reporting Summary

Nature Portfolio wishes to improve the reproducibility of the work that we publish. This form provides structure for consistency and transparency in reporting. For further information on Nature Portfolio policies, see our Editorial Policies and the Editorial Policy Checklist.

Statistics

For all statistical analyses, confirm that the following items are present in the figure legend, table legend, main text, or Methods section.

n/a	Confirmed	
☐	☒	The exact sample size (*n*) for each experimental group/condition, given as a discrete number and unit of measurement
☐	☒	A statement on whether measurements were taken from distinct samples or whether the same sample was measured repeatedly
☐	☒	The statistical test(s) used AND whether they are one- or two-sided<br>*Only common tests should be described solely by name; describe more complex techniques in the Methods section.*
☐	☒	A description of all covariates tested
☐	☒	A description of any assumptions or corrections, such as tests of normality and adjustment for multiple comparisons
☐	☒	A full description of the statistical parameters including central tendency (e.g. means) or other basic estimates (e.g. regression coefficient) AND variation (e.g. standard deviation) or associated estimates of uncertainty (e.g. confidence intervals)
☐	☒	For null hypothesis testing, the test statistic (e.g. *F*, *t*, *r*) with confidence intervals, effect sizes, degrees of freedom and *P* value noted<br>*Give P values as exact values whenever suitable.*
☒	☐	For Bayesian analysis, information on the choice of priors and Markov chain Monte Carlo settings
☒	☐	For hierarchical and complex designs, identification of the appropriate level for tests and full reporting of outcomes
☐	☒	Estimates of effect sizes (e.g. Cohen's *d*, Pearson's *r*), indicating how they were calculated

Our web collection on statistics for biologists contains articles on many of the points above.

Software and code

Policy information about availability of computer code

Data collection	NCBI BLAST 2.9.0
Data analysis	l0x Genomics Cell Ranger 6.0.0, Seurat v2.3.3 R package, rsvd 1.0.2 R package, ModularityOptimizer 1.3.0, singleCellHaystack 0.3.4, NCBI BLAST 2.9.0, OrthoFinder 2.3.1, nlme 3.1-153 R 4.3.1 package, ape 5.6 R 4.3.1 package, rcompanion 2.4.15 R 4.3.1 package, MAFFT 7.4.90, HyPh-RELAX, Pacific Biosciences SMRT LINK V11, lima 2.7.1 Python 3.9.16 package, lsoSeq3 3.8.2 Python 3.9.16 package, minimap2 2.22-r1110-dirty, NCBI lgBLAST 1.17.1, CD-HIT, GUIDENCE2, simboot 0.2.6 R 4.3.1 package, mclogit_0.9.8 R 4.3.1 package, scanpy 1.9.5 Python 3.9.16 package, scirpy 0.13.1 Python 3.9.16 package. Code to build the data resources and to reproduce the figures: https://zenodo.org/record/8384311

For manuscripts utilizing custom algorithms or software that are central to the research but not yet described in published literature, software must be made available to editors and reviewers. We strongly encourage code deposition in a community repository (e.g. GitHub). See the Nature Portfolio guidelines for submitting code & software for further information.

Data

Policy information about availability of data

All manuscripts must include a data availability statement. This statement should provide the following information, where applicable:

- Accession codes, unique identifiers, or web links for publicly available datasets
- A description of any restrictions on data availability
- For clinical datasets or third party data, please ensure that the statement adheres to our policy

Raw fastq files, filtered (empty barcodes and sparse genes) gene-by-barcode UMI count comma separated files, and aligned read BAM files, are available at the Gene Expression Omnibus (GEO), under accession: GSE214390 [https://www.ncbi.nlm.nih.gov/geo/query/acc.cgi?&acc=GSE214390]. All other data produced and used in this work are provided as Supplementary Tables and Files.

Research involving human participants, their data, or biological material

Policy information about studies with human participants or human data. See also policy information about sex, gender (identity/presentation), and sexual orientation and race, ethnicity and racism.

Reporting on sex and gender	N/A
Reporting on race, ethnicity, or other socially relevant groupings	N/A
Population characteristics	N/A
Recruitment	N/A
Ethics oversight	N/A

Note that full information on the approval of the study protocol must also be provided in the manuscript.

Field-specific reporting

Please select the one below that is the best fit for your research. If you are not sure, read the appropriate sections before making your selection.

☒ Life sciences ☐ Behavioural & social sciences ☐ Ecological, evolutionary & environmental sciences

For a reference copy of the document with all sections, see nature.com/documents/nr-reporting-summary-flat.pdf

Life sciences study design

All studies must disclose on these points even when the disclosure is negative.

Sample size	Sample sizes were determined based on several factors:<br>l. Sampling animals from several distinct naked mole-rat colonies<br>2. Sampling animals from distinct age groups (where relevant)<br>3. Sampling animals from distinct sexes (where relevant)<br>4. Keeping samples sizes sufficiently large for obtaining sufficient sequencing coverage<br>5. Keeping samples sizes consistent with best practices in the field, which is a minimum of 3 samples (animals) for each group (species, and where relevant age and sex)
Data exclusions	A single naked mole-rat spleen T-cell scRNA-seq sample was disqualified due to poor RNA quality discovered post sequencing
Replication	1. Considerations of our sample sizes<br>2. Sampling different immune compartments in order to test consistency of our results<br>3. Using different data modalities (scRNA sequencing and genome assemblies) to support findings in our work
Randomization	1. All animals were randomly sampled and/or ordered<br>2. Tissue processing was conducted randomly WRT to the experimental covariates in order to avoid batch effects<br>3. Sequencing of all RNA samples was spread across all sequencing lanes in order to avoid batch effects
Blinding	Blinding was not possible, neither in the selection of samples nor in their analysis because covariates (such as age and sex) were required to be known prior to sample selection and analysis.

Reporting for specific materials, systems and methods

We require information from authors about some types of materials, experimental systems and methods used in many studies. Here, indicate whether each material, system or method listed is relevant to your study. If you are not sure if a list item applies to your research, read the appropriate section before selecting a response.

Materials & experimental systems

n/a	Involved in the study
☐	☒ Antibodies
☒	☐ Eukaryotic cell lines
☒	☐ Palaeontology and archaeology
☐	☒ Animals and other organisms
☒	☐ Clinical data
☒	☐ Dual use research of concern
☒	☐ Plants

Methods

n/a	Involved in the study
☒	☐ ChIP-seq
☐	☒ Flow cytometry
☒	☐ MRI-based neuroimaging

Antibodies

Antibodies used

1. Anti-naked-mole-rat CD3e. Abbvie-clone-5, Alexa 647, 10 ug/ml.
2. Anti-mouse CDllb. clone Ml/70, eBioscience™, (#12-0112-82), 2 and 5 ug/ml, for mice and NMRs, respectively
3. Anti-mouse CD3e. clone 17A2, eFlour450, eBioscience™, (#48-0032-82), 2 ug/ml.

Validation

The anti-naked-mole-rat CD3e antibody was validated in-house using a series of dilutions of the florescence-conjugated antibody and measuring the resulting percentage of CD11b-/CD3e+ sorted cells.

Animals and other research organisms

Policy information about studies involving animals; ARRIVE guidelines recommended for reporting animal research, and Sex and Gender in Research

Laboratory animals

C57BL/6 mice, purchased from Jackson Laboratories (Bar Harbor, ME), JAX stock #000664, (2 months of age, referred to as "adult" throughout the text and figures; 24 months of age, referred to as "old" throughout the text and figures; all virgins), housed within the Laboratory Animal Resources (LAR) vivarium, which is part of the AAALAC-accredited animal care and use program at the Calico Life Sciences LLC, at a room temperature range of 14.5-26C with a humidity range of 30-70%, maintained on a 12-house dark-light cycle, receiving food and water ad libitum, and used in experiments only after two weeks of vivarium housing.. NMRs (2 years of age, referred to as "adult" throughout the text and figures; 26-28 years of age, referred to as "old" throughout the text and figures; all non-breeding virgins), from 20 different captive colonies housed within Calico Life Sciences colonies at the Buck Institute, Novato, CA, at a room temperature range of 28-31C with a humidity range of 40-50%, maintained on a 12-house dark-light cycle, receiving food ad libitum yet no supplemented water since the water content of their fresh fruit and vegetable diet is sufficient for maintaining appropriate
hydration, in accordance with standard colony management.

Wild animals

No wild animals were used in this work.

Reporting on sex

In all studies conducted in this work, except for the sequencing of splenic T cells and their receptors, both sexes of mouse and NMR were used, hence we believe that our results are representative of both sexes.

Field-collected samples

No field samples were used in this work

Ethics oversight

All animal use and experiments were approved by the Buck Institute institutional animal care and use committee (IACUC) protocol number A10173.

Note that full information on the approval of the study protocol must also be provided in the manuscript.

Plants

Seed stocks

Report on the source of all seed stocks or other plant material used. If applicable, state the seed stock centre and catalogue number. If plant specimens were collected from the field, describe the collection location, date and sampling procedures.

Novel plant genotypes

Describe the methods by which all novel plant genotypes were produced. This includes those generated by transgenic approaches, gene editing, chemical/radiation-based mutagenesis and hybridization. For transgenic lines, describe the transformation method, the number of independent lines analyzed and the generation upon which experiments were performed. For gene-edited lines, describe the editor used, the endogenous sequence targeted for editing, the targeting guide RNA sequence (if applicable) and how the editor was applied.

Authentication

Describe any authentication procedures for each seed stock used or novel genotype generated. Describe any experiments used to assess the effect of a mutation and, where applicable, how potential secondary effects (e.g. second site T-DNA insertions, mosiacism, off-target gene editing) were examined.

Flow Cytometry

Plots

Confirm that:

- [ ] The axis labels state the marker and fluorochrome used (e.g. CD4-FITC).
- [ ] The axis scales are clearly visible. Include numbers along axes only for bottom left plot of group (a 'group' is an analysis of identical markers).
- [ ] All plots are contour plots with outliers or pseudocolor plots.
- [ ] A numerical value for number of cells or percentage (with statistics) is provided.

Methodology

Sample preparation	Mouse splenocytes were pre-incubated with 1ug mouse Fc blocker (BD 553141) per 1 million cells/100 ul FACS buffer for 5 minutes and incubated with an anti-mouse CD3e (clone 17A2, eFlour450, 2ug/ml) and an anti-mouse CD11b (M1/70, Alexa 488, 2 ug/ml) on ice for 40 minutes.<br>NMR splenocyes were pre-incubated with the isotype control antibodies (100 ug/ml migG1 & 50 ug/ml rat IgG1) per 1 million cells /100 u; FACS buffer for 10 minutes. Subsequently, cells were washed and resuspended at 2 million cells per 1ml FACS buffer and incubated with an anti-NMR CD3e (Abbvie-clone-5, Alexa 647, 1:100) and an anti-mouse CD11b (which cross reacts with the NMR CD11b83) (M1/70, R-PE, 5 ug/ml) on ice for 40 minutes.<br>For both mouse and NMR cells, after incubation cells were washed and resuspended in the FACS buffer containing Sytox Blue Live/Dead dye for cell sorting using FACS BD Aria.
Instrument	FACS BD Aria
Software	*Describe the software used to collect and analyze the flow cytometry data. For custom code that has been deposited into a community repository, provide accession details.*
Cell population abundance	*Describe the abundance of the relevant cell populations within post-sort fractions, providing details on the purity of the samples and how it was determined.*
Gating strategy	*Describe the gating strategy used for all relevant experiments, specifying the preliminary FSC/SSC gates of the starting cell population, indicating where boundaries between "positive" and "negative" staining cell populations are defined.*

- [ ] Tick this box to confirm that a figure exemplifying the gating strategy is provided in the Supplementary Information.
